# Therapist Experience and Knowledge Acquisition in Internet-Delivered CBT for Social Anxiety Disorder: A Randomized Controlled Trial

**DOI:** 10.1371/journal.pone.0037411

**Published:** 2012-05-23

**Authors:** Gerhard Andersson, Per Carlbring, Tomas Furmark, on behalf of the S. O. F. I. E. Research Group

**Affiliations:** 1 Department of Behavioural Sciences and Learning, Swedish Institute for Disability Research, Linköping University, Linköping, Sweden; 2 Department of Clinical Neuroscience, Psychiatry Section, Karolinska Institutet, Stockholm, Sweden; 3 Department of Psychology, Umeå University, Umeå, Sweden; 4 Department of Psychology, Uppsala University, Uppsala, Sweden; Institute of Psychiatry at the Federal University of Rio de Janeiro, Brazil

## Abstract

**Background:**

Guided internet-delivered cognitive behavior therapy (ICBT) has been tested in several trials on social anxiety disorder (SAD) with moderate to large effects. The aims of this study were threefold. First, to compare the effects of ICBT including online discussion forum with a moderated online discussion forum only. Second, to investigate if knowledge about SAD increased following treatment and third to compare the effects of inexperienced versus experienced therapists on patient outcomes.

**Methods:**

A total of 204 participants with a primary diagnosis of SAD were included and randomized to either guided ICBT or the control condition. ICBT consisted of a 9-week treatment program which was guided by either psychology students at MSc level (n = 6) or by licensed psychologists with previous experience of ICBT (n = 7). A knowledge test dealing with social anxiety was administered before and after treatment. Measures of social anxiety and secondary outcomes dealing with general anxiety, depression, and quality of life were administered before and after treatment. In addition, a 1-year follow-up was conducted on the treated individuals.

**Results:**

Immediately following treatment, the ICBT group showed superior outcome on the Liebowitz Social Anxiety Scale self-report version with a between group posttreatment Hedges *g* effect size of *g* = 0.75. In addition, significant differences on all the secondary outcomes were observed. Gains were well maintained one year later. Knowledge, as assessed by the knowledge test, increased following treatment with little gain in the control group. Therapist experience did not result in different outcomes, but experienced therapists logged in less frequently compared to the inexperienced therapists, suggesting that they needed less time to support patients.

**Discussion:**

We conclude that guided ICBT reduce symptoms of SAD, increase knowledge about SAD and that therapist experience does not make a difference apart from the finding that experienced therapist may require less time to guide patients.

**Trial Registration:**

UMIN.ac.jp UMIN000001383

## Introduction

Internet-based cognitive-behavior therapy (ICBT) was developed in the late 1990’s [1], [2], and has been investigated in a large number of randomized controlled trials as attested by systematic reviews and meta-analyses on anxiety disorders [3], mood disorders [4], and somatic health conditions [5]. Social anxiety disorder (SAD), often referred to as social phobia [6], is a condition for which strong empirical support exists regarding standard CBT provided in groups or individually [7], [8], [9]. There is also some evidence that SAD can be treated with bibliotherapy [10], [11], [12], but more studies have been conducted on ICBT. Indeed, following the first ICBT trial [13], and further later studies conducted by our group [12], [14], [15], [16], [17], the findings have been replicated by three separate research groups with one in Australia [18], [19], [20], [21], [22], [23], and two additional groups in Europe [24], [25], [26], totaling at least 15 controlled trials. Interestingly, stable long-term effects have also been found up to five years after treatment completion [27], [28]. In addition, there is evidence to suggest that the treatment is effective under clinically representative conditions [16], [29], so called effectiveness studies [30], where patients are treated in regular clinical settings with therapists who work there not only as researchers. One topic that has been investigated in several trials is the need for human support in ICBT for SAD. While the overall message from the literature suggests that support in the form of human guidance is needed to generate good outcomes in ICBT [31], [32], this is not necessarily the case in the treatment of SAD if a proper diagnosis has been established [20], [23], [24]. Another question concerns who should provide the support. The variance explained by the therapist factor is small to non-existent in ICBT [33], [34], and Titov et al. have found that support can be provided mainly from a practical and technical point of view [21], [35], [36]. However, a potential problem with the above mentioned trials is that few therapist have been involved and hence it is not known if inexperienced and experienced ICBT-therapists differ in terms of effects and time needed to give the support in ICBT.

Even if CBT always include psychoeduction there are very few trials in which knowledge acquisition has been directly addressed. Indeed, the treatment sessions in ICBT are often referred to as lessons [19], or as modules [37], making it motivated to ask about knowledge acquisition. One exception is a study by Scogin et al. [38], in which a test of knowledge regarding depression was administered before and after bibliotherapy for depression. While knowledge increased in that study, the knowledge gained was not related to improvement in depressive symptoms. On the other hand, a more recent study found that knowledge about affective disorders and treatment was predictive of outcome two years later in a study on depression [39].

We had three aims of this study. First, we wanted to compare guided ICBT for SAD with a moderated online discussion group instead of a pure waitlist control group. There is conflicting evidence regarding the general effectiveness of online support groups [40], [41]. However, using the Internet has been perceived as helpful for some persons with SAD [42], and there are persons who benefit from getting support from others in the same situation and even advice regarding treatment, including therapeutic advice on how to handle anxiety. Moreover, participation in an online discussion group is distinctly different from pure waiting. In line with the previous results we expected guided ICBT to be superior to participation in a moderated discussion group. Second, we investigated if knowledge about SAD and its treatment components was influenced by our treatment. We hypothesized that knowledge would increase but we did not have a clear hypothesis regarding the correlation between knowledge and clinical improvement. Third, we wanted to randomly assign participants to either inexperienced or experienced therapists from whom support was provided during the treatment. Again we did not have a clear hypothesis, as previous studies have not found any major differences between categories of therapist/support persons in guided ICBT. However, we also investigated the number of log ins (to a secure messaging system) needed to give the support, as we expected that experienced therapists would spend less time with their patients.

## Methods

### Trial Design

The study protocol and supporting CONSORT checklist for this trial are available as supporting information; see Checklist S1 and Protocol S1. This was a superiority trial within the context of a parallel group study with blocked randomization in 1∶1 ratio. Outcome assessors were blind to treatment status.

### Ethics

The trial was approved by the regional ethics committee at Uppsala University, Sweden. The trial is registered at University Hospital Medical Information Network (http://www.umin.ac.jp/) UMIN000001383.

### Recruitment and Selection

The recruitment procedure was largely similar to our previous studies on ICBT for SAD [12], [13], [14]. Participants were recruited via a research web page (www.studie.nu), which had been advertised by mass media in Sweden. A web portal including online questionnaires and a secure email service was used in the trial. The system handles security issues with two factor authentication in order to decrease the probability that the requestor is presenting false evidence of its identity. On the study web page, information about the trial was presented. Potential participants were advised to complete an application form and an online screening battery consisting of the Social Phobia Screening Questionnaire (SPSQ) [43], the self-rated version of the Montgomery Åsberg Depression Rating Scale (MADRS-S) [44], and additional questions regarding current and past treatments including medication. Persons who passed this initial step were contacted for a telephone interview, and if not suitable given advice and a reason why they were not included. To be included, they had to meet the following criteria: (a) a DSM-IV [6] diagnosis of SAD according to the SPSQ; (b) scoring <31 on the MADRS-S depression scale and <4 on the suicide item of this scale to prevent the inclusion of individuals in strong need of specialist consultation; (c) not undergoing any other psychological treatment during the study period; (d) if on prescribed medication for anxiety/depression, dosage had to be constant for 3 months before the treatment onset and kept constant throughout the study; (e) being at least 18 years old; (f) living in Sweden; (g) having access to a computer with Internet connection; (h) not admitting another serious or dominant disorder (e.g. psychosis, substance misuse) that could be expected to influence the outcome of the study; and (i) a primary diagnosis of SAD according to the Structured Clinical Interview for DSM-IV, SCID-I [45]. The last criterion was evaluated by a telephone-interview in which the diagnostic questions from the SAD section of the SCID-I were posed. When a person failed to meet the inclusion criteria an individual electronic message was sent with advice on where to seek more appropriate help.

**Figure 1:**
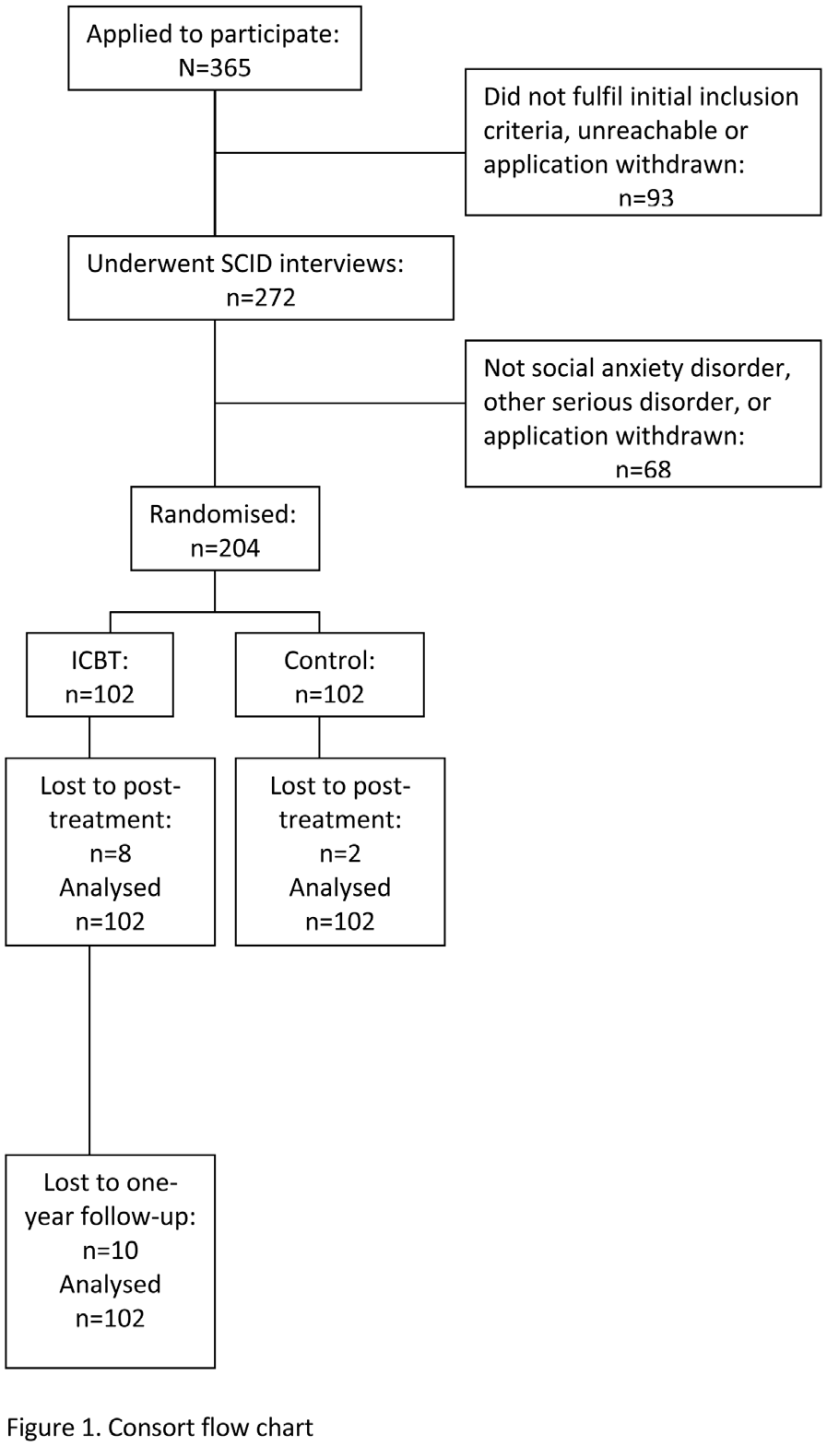
CONSORT flow chart.

Of the 365 individuals who applied to participate, 204 individuals meeting all inclusion criteria were eventually randomized to either treatment (n = 102) or a control condition (n = 102). Randomization was performed by an independent third-party using an online true random-number service (www.random.org). The control group received delayed treatment after 9 weeks and the outcome of their treatment will not be reported here. Eight participants in the treatment group and 2 in the control group did not complete posttreatment data yielding a 5% dropout. In accordance with the intention-to-treat principle, all participants were asked to complete posttreatment and follow-up assessments, regardless of how many treatment modules they had completed and all were included in the analyses. One-year follow-up data were not provided by 10 subjects in the treatment group (10.6%). Written informed consent was obtained. An overview of the procedure is given in Figure 1. Patient characteristics are presented in Table 1.

**Table 1:** Descriptive characteristics.

	ICBT (n = 102)	Control (n = 102)
Gender: Female, n (%)	62 (77.5)	61 (60.0)
Age: years, M (SD)	38.1 (11.3)	38.4 (10.9)
Range	19–66	19–71
Married or de facto, n (%)	66 (64.7)	66 (64.7)
Employment status: full-time, n (%)	78 (76.5)	73 (71.6)
student, n (%)	14 (13.7)	16 (15.7)
Not in work/retired/unemployed, n %	10 (9.8)	13 (12.7)
Prescription:ongoing medication, n (%)previous medication, n (%)	10 (9.8)41 (40.2)	18 (17.6)30 (29.4)
Education: College/University n (%)	43 (42.1)	55 (53.9)
Had earlier psychologicaltreatment, n (%)	53 (51.9)	59 (57.8)
Generalized subtype, n (%)	63 (61.7)	66 (64.7)

### Outcome Measures

#### Social anxiety

Four social anxiety questionnaires were used as outcome measures: the Liebowitz Social Anxiety Scale self-report version (LSAS-SR) [46], [47], the Social Phobia Scale (SPS) [48], the Social Interaction Anxiety Scale (SIAS) [48], and the SPSQ [49]. We regarded the LSAS-SR as the primary outcome measure [50]. In addition to the questionnaires administered at pre-, post-, and follow-up assessments, participants in the treatment group completed the LSAS-SR online every week (Sundays). In case of missing data, a brief and neutral reminder was sent 24 hours later via e-mail, and if necessary, followed by another reminder sent as an auto-generated short-text-message (SMS) to the person’s mobile phone. The response rate on these weekly assessments ranged between 80–100% across modules and the last previous LSAS-SR score was used to replace missing data.

#### General anxiety, depression and quality of life

Three secondary measures were used to measure general anxiety, depression and quality of life: the Beck Anxiety Inventory (BAI) [51], the MADRS-S [52], and the Quality of Life Inventory (QOLI) [53].

#### Knowledge test

We developed a knowledge test dealing with the condition of SAD and its treatment. The test construction involved selection of test items, consulting experts in the field of knowledge tests and social anxiety, and pilot testing with psychology students (n = 24) and a second pilot testing (n = 18 students) after revisions based on feedback from students and experts. The final test included 11 items with multiple choice response options (three options). In addition, each response was rated in terms of how certain the participant was about the response with three response options (Guessing, Pretty certain, Confident or convinced). A higher score indicate more knowledge. Items included were: 1. What is the core problem in SAD; 2. One important rule in treatment of phobias is…; 3. What is a safety behavior?; 4. Which one of these alternatives is a typical automatic thought?; 5. Which one of these alternatives characterizes a thought trap?; 6. What is the most important reason for defining treatment goals in CBT?; 7. What is a core belief according to CBT?; 8. What is exposure?; 9. One component in CBT for SAD is called “shifting focus”, what does that stand for?; 10. Which technique is recommended in CBT if you want to express your dissatisfaction to someone?; 11. Which one of these alternatives is the main reason why it can be difficult to get rid of SAD? We scored the knowledge test in two ways. First we calculated a total score based on total number of correct answers. Second we calculated a weighted total score in which certainty of answers were factored in. Basically this meant a higher score if you were correct and certain, a lower score if you were right, yet uncertain, and finally a negative score if you were wrong but certain. Reliability analyses showed a low Cronbach́s alpha of α = .40 for the raw scores, a high Cronbach́s alpha of α = .86 for the certainty ratings, and an alpha of α = .56 for the weighted scores.

### Administration of Self-report Measures

All self-report instruments were administrated via the Internet at pretreatment (baseline), posttreatment and one-year follow up. Adequate psychometric properties have previously been demonstrated for Internet-administered questionnaires relating to SAD [54], with Cronbach́s alpha values ranging between α = .89 to α = .94 for the SAD measures, and α = .81 to α = .89 for the secondary outcome measures used in this study.

### Global Functioning and Improvement

A telephone interview was conducted using the Clinical Global Impression Improvement Scale (CGI-I) [55] to measure global improvement after the treatment period. Outcome assessors were not aware of treatment status before the interview.

### Treatment Procedure

All participants had to have access to a computer with an Internet connection, a web browser and the ability to print out files in PDF format. Participants were recommended to get a free online e-mail service that automatically encrypts messages in 2048 bits instead of using their personal e-mail. However, this was just used for reminders to log in to the secure messaging system.

The main treatment component was our previously evaluated self-help manual for SAD [56], which consists of 186 pages divided into nine chapters (modules) adapted for use via the Word Wide Web. In brief, the manual starts with an introductory module describing SAD and facts about CBT. Modules 2–4 describe a cognitive model for SAD and introduce cognitive restructuring. Modules 5–7 introduce exposure and attention shifting exercises. Modules 8–9 mainly concern social skills and relapse prevention. Participants were asked to complete one module every week, i.e. a 9-week treatment period was recommended. Each module consisted of information, exercises (home-work assignments) and ended with a short quiz to check adherence. Feedback was given each week by the therapists. Text messages were used to remind participants to log in or to complete weekly reports on the LSAS-SR.

### Online Discussion Forum

Participants in both groups had access to separate moderated online discussion forums. For each week, participants were asked to post at least one message in the discussion group about a new but predetermined topic. Discussions were monitored but the study personnel did not take active part in them unless it was needed. The study team posted the topics for discussion (e.g., “What are your experiences of seeking help for SAD”), and were ready to comment if questions were asked directly to the study team or if discussions would be seen as inappropriate (e.g., negative comments about other participants, expression of suicidal intent, etc). The latter did not occur. The discussion group was open during the whole study period. Directly following the waiting period for the online discussion group, the participants commenced the same Internet-delivered self-help treatment as for the ICBT group.

### Internet Therapists

The treatment group had access to an Internet therapist during the 9 week treatment period. E-mail correspondence occurred weekly (Sundays) and generally concerned the results of homework assignments as described in the self-help manual. The rationale behind the homework assignments was to promote learning and enable the Internet therapists to decide whether the participants had assimilated the information and completed their exercises. In general, therapist feedback on the homework assignment was provided within 24 hours after the participant had submitted a massage. We collected data on how often the therapist logged in, which is a proxy of how much time they devoted to the participant. Participants could ask questions all week and receive a response, but feedback on progress was mainly given after the Sunday deadline. We did not register how long the therapists were in the system, but experienced therapists were more likely to handle all participants once they were in the system, whereas inexperienced therapists (who were students) had more time to check the system frequently and spend more time on each participant. Moreover, therapists were instructed to only be in the system when responding and giving feedback, and not stay in the system when doing other things. When the homework was completed, the next module was made accessible. Alternatively, an instruction on what needed to be completed to proceed to the next module was sent to the participant. On average, Internet therapists spent 15 min per week for each participant reading messages and providing feedback via the online contact handling system.

There were 13 Internet-therapists in the study, 7 of which were licensed clinical psychologists with an average or 3 years of clinical experience (range 2–6 years), and 6 clinical psychology students in their last semester of the five years master’s degree program. All therapists had completed a basic training in CBT including supervised face-to-face therapies. The students (i.e., inexperienced therapists) had clinical supervision during the trial. Participants were randomly allocated to their therapist, with the restriction that each therapist only could be allocated a limited number of participants. The experienced therapists had worked with previous Internet trials on anxiety and mood disorders. There were four men and three women among the experienced therapists and one man and five women among the student therapists. The average age of the experienced therapists was M = 28.9 years (SD = 2.4). The corresponding age among the student therapists was M = 29.6years (SD = 9.0; mainly caused by one of the student therapist being older). The student therapists treated 60 participants (10 each) and the experienced therapists 42 participants (6 each).

### Sample Size and Power

The trial was originally powered to investigate genetic effects (see Protocol S1), and we aimed to include 250 participants, with 125 in each arm. However, we were not able to recruit the full sample which required consent to genetic testing. For the present report and in light of the previous studies on ICBT for SAD (see introduction), a moderate between group effect size of *g* = 0.50 would require at least 32 participants in each group, with an alpha at 05 and a power of 80%. The study was thus sufficiently powered for detecting treatment effects. The power for the difference between experienced and inexperienced therapist could not be based on previous studies, and if we assume a small difference (*g* = 0.20), a much larger sample would have been required (at least 160 in each group).

### Statistical Analyses and Clinical Significance

We used a mixed models approach to analyze the data with full information maximum likelihood estimation [57]. Linear mixed effect models are able to accommodate missing data and integrate time-varying factors. It has been recommended to use mixed models analyses as a way to handle intention to treat data [58]. However, one assumption is that the lost data is missing at random and not non-ignorable. We made this assumption as no obvious pattern was observed for the missing data and the actual loss of data was relatively small. We used an unstructured covariance structure for the analyses. An unstructured covariance structure has the property which means that the correlations between measurements at different time points are allowed to vary. Hence, the correlation between the Pre vs. Post measurement and post vs. 1-year follow-up did not necessarily have to be constant.

Clinically significant improvement was determined for the LSAS-SR in accordance with Jacobson and Truax criteria [59] by using the Reliable Change Index for each individual and a post-test score within two standard deviations (SDs) of the mean of the normal population [47]. Chi-square was used to test distribution differences with regard to clinically significant improvement and demographic/descriptive characteristics.

## Results

### Randomization Check, Attrition and Treatment Completion

There were no statistically significant differences between the treatment and the control groups with regards to demographics (Table 1) or pretreatment self-report scores (Tables 2 and 3). The average number of completed modules in the treatment group was 6.8 (SD = 3.07) out of a total of nine. In total there were 46/102 (45%) participants who failed to complete all nine modules during the nine week treatment period. The activity in the online discussion forums varied in line with previous investigations [12], which means that a majority made few comments and postings in addition to the postings linked to the “topic of the week”, some were more active with discussions, and some mainly read were passive.

**Table 2:** Immediate results with time x group interaction and estimated means and standard error at pre and post (n = 204) in accordance with the Intention-to-treat principle.

	Treatment			Control group			Interaction
	M	SE	SD	M	SE	SD	(F)
**Liebowitz Social Anxiety Scale Self-Report Version**							
Pre	68.23	2.23	23.33	66.65	2.23	21.72	95.62***
Post	43.74	2.42	24.33	63.85	2.40	23.69	
**Social Phobia Scale**							
Pre	38.81	1.51	15.59	37.25	1.51	14.98	50.90***
Post	23.31	1.46	14.33	32.90	1.44	14.76	
							
**Social Interaction Anxiety Scale**							
Pre	49.96	1.54	15.88	48.88	1.54	15.28	68.43***
Post	33.77	1.52	15.33	46.02	1.49	14.67	
**Beck Anxiety Inventory**							
Pre	15.73	0.85	7.98	16.47	0.85	9.14	19.20***
Post	9.46	0.75	6.42	14.00	0.74	8.35	
							
**Montgomery Åsberg Depression Rating Scale (Self-Rating Version)**							
Pre	13.45	0.68	7.14	14.29	0.68	6.63	22.56***
Post	9.90	0.73	7.23	14.75	0.72	7.20	
**Quality of Life Inventory**							
Pre	0.65	0.18	1.86	0.58	0.18	1.68	7.67**
Post	1.29	0.19	2.04	0.76	0.19	1.69	
**Social Phobia Screening Questionnaire**							
Pre	30.77	0.89	8.94	30.54	0.89	9.07	47.51***
Post	18.93	1.01	10.42	26.76	1.00	9.65	

In addition, to facilitate the understanding the observed standard deviation was added (n = 204 and 195 at pre and post respectively).

Note: ** = p<.01; *** = p<.001.

**Table 3:** Results of the test of specific knowledge pre vs. post treatment in the two groups (n = 197).

	Treatment			Control			Interaction
	M	SE	SD	M	SE	SD	(F)
**Test of specific knowledge (unweighted)**							
Pre	7.32	0.18	1.83	7.45	0.17	1.63	40.04***
Post	8.81	0.18	1.70	7.53	0.17	1.78	
**Test of specific knowledge (weighted)**							
Pre	8.65	0.75	7.49	8.87	0.75	7.37	86.52***
Post	18.63	0.83	8.73	9.70	0.82	7.61	

*** = p<.001.

## Treatment Effects – Primary Outcome

**Figure 2:**
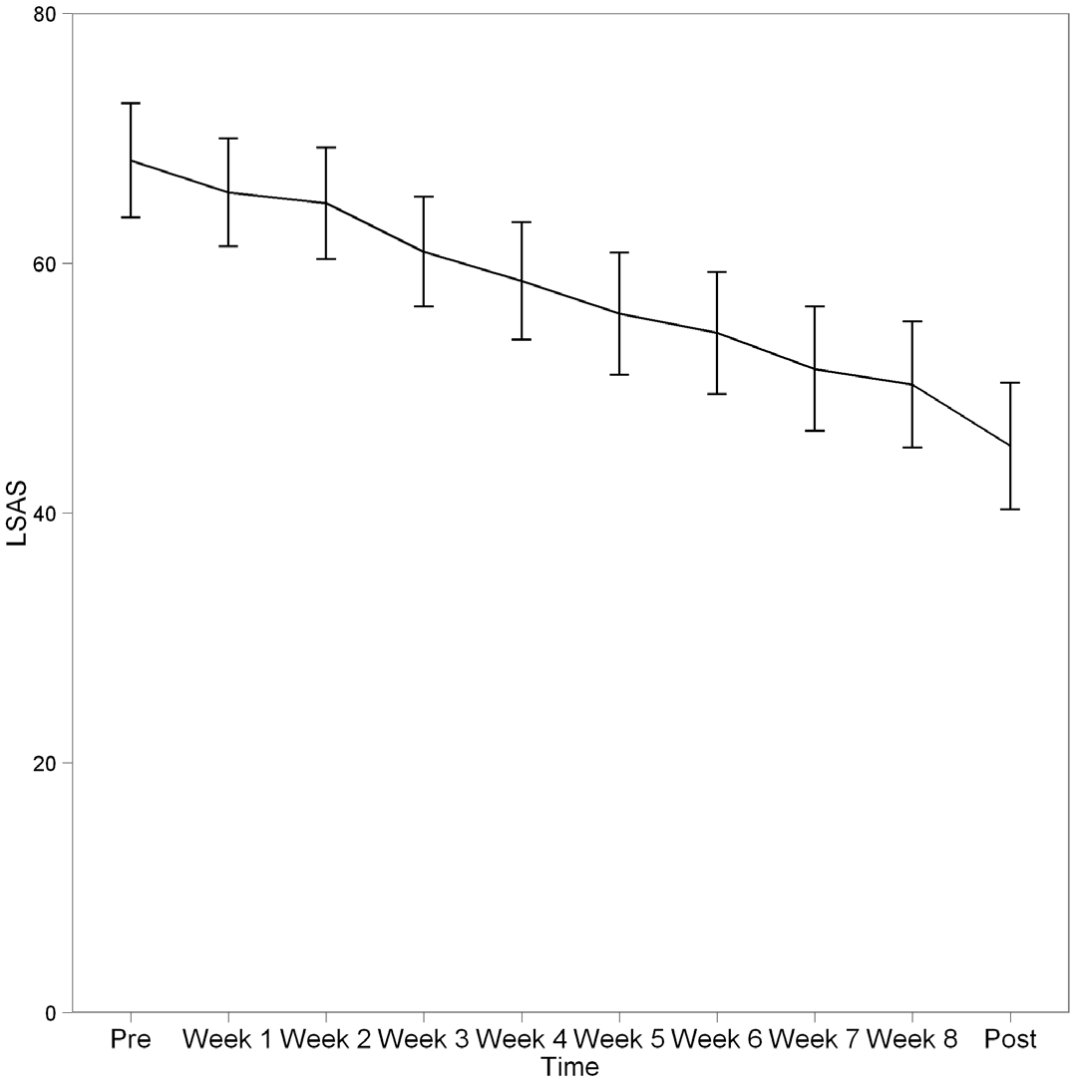
Weekly measures of Liebowitz Social Anxiety Scale self-report version (LSAS-SR) for the treatment group.

On the main outcome measure LSAS-SR, a large interaction effect was identified (Table 2), with a between group posttreatment Hedges *g* effect size of *g* = 0.75 (95% CI = 0.46–1.03). Results for the weekly measurement points are presented in Figure 2 for the treatment group. A linear trend was seen as a decrease in symptoms over the weeks in treatment. One year follow-up data were collected for the treatment group. On the LSAS-SR scores remained improved compared to pretreatment (M = 40.39, SD = 23.6). The change from posttreatment to one year follow-up was in the direction of further improvement, albeit not significant (*t*_89_ = 1.92, *p* = .058).

The number of participants meeting the criteria for clinically significant improvement was calculated for the LSAS-SR. In the treatment group, 45.1% (n = 46/102; CI 95% 35.3%–54.9%) reached this criterion versus 11.8% (n = 12/102; CI 95% 5.4%–18.1%) in the online discussion forum control group. The difference was statistically significant *χ*2(1) = 27.8, *p*<.001.

## Treatment Effects – Secondary Outcomes

As evident from Table 2, statistically significant interactions were found for the three additional measures of social anxiety symptoms; the SPS (p<.001), the SIAS (p<.001), and the SPSQ (p<.001). Improvements, as evidenced by significant interaction effects, were also identified on measures of general anxiety (BAI, p<.001), depression (MADRS-S, p<.001), and quality of life (QOLI, p<.01).

Between group effect sizes at posttreatment ranged between a low of g = 0.25 for the QOLI and a high of g = 0.72 for SIAS. One year follow-up data for these measures are presented in Table 4 (separated for the two types of therapist experience). On all secondary outcome measures the changes obtained were retained or slightly improved at follow-up.

**Table 4:** Immediate and 1 year follow-up results of the relative effect of therapist experience in accordance with the Intention-to-treat principle (n = 102).

	Experienced			Inexperienced			Interaction (F)	Pairwise Comparison
	M	SE	SD	M	SE	SD		
**Liebowitz Social Anxiety Scale Self-Report Version**								
Pre	67.95	3.62	26.47	68.42	3.03	21.10	0.40	Pre<Post = Fup
Post	42.30	3.86	25.91	44.80	3.21	23.36		
Follow-up	41.02	3.80	25.13	41.44	3.13	22.74		
**Social Phobia Scale**								
Pre	39.98	2.41	17.58	38.00	2.02	14.12	0.60	Pre<Post<Fup
Post	23.97	2.25	14.11	22.79	1.88	14.60		
Follow-up	20.95	2.38	15.62	21.51	1.96	14.73		
**Social Interaction Anxiety Scale**								
Pre	50.17	2.46	17.66	49.82	2.06	14.66	0.46	Pre<Post<Fup
Post	34.00	2.43	15.69	33.99	2.03	15.22		
Follow-up	30.98	2.51	16.61	32.49	2.07	15.20		
**Beck Anxiety Inventory**								
Pre	15.50	1.24	8.89	15.88	1.03	7.35	0.52	Pre<Post<Fup
Post	9.74	1.00	7.41	9.19	0.84	5.67		
Follow-up	8.03	1.06	6.76	8.45	0.87	6.54		
**Montgomery Åsberg Depression Rating Scale (Self-Rating Version)**								
Pre	13.19	1.11	7.78	13.63	0.93	6.71	2.15	Pre<Post = Fup
Post	10.13	1.14	7.98	9.75	0.95	6.70		
Follow-up	8.14	1.17	6.85	10.15	0.96	7.45		
**Quality of Life Inventory**								
Pre	0.58	0.29	2.02	0.71	0.24	1.75	0.73	Pre<Post = Fup
Post	1.36	0.32	2.27	1.20	0.27	1.88		
Follow-up	1.49	0.29	1.95	1.50	0.24	1.69		
**Social Phobia Screening Questionnaire**								
Pre	31.36	1.38	10.07	30.37	1.16	8.12	0.72	Pre<Post = Fup
Post	19.52	1.64	10.98	18.55	1.37	10.08		
Follow-up	17.55	1.71	10.64	18.17	1.41	10.52		

In addition, to facilitate the understanding the observed standard deviation was added (with n = 102, 95 and 92 at pre, post and follow-up respectively). Experienced therapists handled 42 participants and inexperienced 60 participants.

## Treatment Effects – Knowledge Test

Results for the knowledge test are presented in Table 3. Both the raw scores and the weighted scores improved following ICBT, but not in the control group (both interactions highly significant). As can be inferred from Table 3, the treatment group became more certain of their answers to the questions. Improvement on the weighted knowledge test was not associated with improvement on the primary outcome measure LSAS-SR, but did correlate with improvement on the SPSQ (Pearson’s r = .26, p = .01) and on the SPS (Pearson’s r = .23, p = .03).

## Clinical Interview

At post-treatment, 36 participants (35.3%) in the treatment group were classified as very much improved or much improved according to the independent CGI-I (95% CI, 25.9%–44.7%). In the control group, the corresponding number of participants was 6 (14.3%) as assessed by the CGI-I (95% CI, 0.6%–9.2%). The difference was statistically significant *χ*2(1) = 26.9, *p*<.001. Adverse events, defined as a CGI-I score of 5 (minimally worse) was found in one case in the treatment group and six cases in the control group. The deteriorations observed could not be linked to the treatment and were rather a worsening of their SAD and related problems.

## Effects of Therapist Experience

The third aim of the study was to investigate if previous therapist experience would make a difference. Results are presented in Table 4. There were no statistically significant interactions suggesting that the two categories of therapist experience produced equal outcomes. On the primary outcome measure LSAS-SR the within-group pre-post Hedges g effect size was g = 0.98 (CI 95% 0.56–1.39) for the experienced group and g = 1.06 (CI 95% 0.63–1.47) for the inexperienced group, yielding very similar outcomes. However, experienced therapists logged in less frequently. Mean number of log-ins was 25.5 (SD = 12.5) as compared to the inexperienced therapists 33.01 (SD = 13.4). This difference was statistically significant (t_100_ = 2.84, p = .005).

## Discussion

This trial investigated the effects of ICBT for persons with a DSM-IV diagnosis of SAD and compared the effects of treatment with being part of an online discussion forum. The trial also investigated if knowledge about social anxiety increased with treatment and if novice and experienced therapists were equally effective when guiding the treatment.

### Is ICBT for SAD Effective?

The first aim concerned testing guided ICBT against a discussion forum control group. Results clearly showed that the active treatment was superior. The between group effect size was large (*g* = 0.75), and in line with a range of previous studies on guided ICBT for SAD [60]. Moreover, the findings are consistent with a range of previous studies showing that guided ICBT is an effective treatment for anxiety disorders and that it can be equally effective as face-to-face CBT [16], [61], [62]. We also found that the effects were sustained at one year follow-up, which is in line with previous studies [27], [28].

### Does Knowledge Increase Following ICBT?

The second aim was to determine if ICBT increases knowledge about SAD and its treatment. To be able to answer this question a new test was developed. Knowledge increased in ICBT but not in controls. Small but statistically significant associations between knowledge gain and outcome were found. There is a surprising scarcity of CBT studies investigating knowledge gains, even if there are studies on mental health literacy [63]. This lack of knowledge calls for more research given the role of psychoeducation in CBT in general and in ICBT and bibliotherapy in particular, as the latter involve reading text material and grasping instructions presented over the internet or in text.

### Does it Matter if the Therapist is Experienced?

The third aim was to investigate the effects of therapist experience. While there are previous studies suggesting that guidance can be given from a practical and technical point of view [21], [35], [36], this is the first and probably the largest ICBT study for SAD in which therapists with less or more previous experience of ICBT have been randomly allocated to patients. The findings suggest that ICBT does not require experienced therapists to be effective. However, it should be noted that all therapists had been trained in CBT. Moreover, the highly structured ICBT protocol leave less room for therapist effects and it is possible that therapist experience would have been more important in a less structured treatment. However, in a previous trial by our group on depression, inexperienced therapist were found to be effective when the treatment was in the form of e-mail and not structured in advance [64]. Interestingly, the more experienced therapist logged in less often which means they spent less time following the participants. There are suggestions that experienced therapist may drift from treatment protocols [65], but is probably less likely to make a difference in ICBT as the treatment content remains the same.

### Limitations

The first limitation of the present study was that we recruited a sample via advertisements and not from a clinic. This limits the generalizability of the findings even if previous studies have indicated that ICBT for SAD is effective in regular clinical settings with similar outcomes as in studies with recruitment from the general public [16], [29]. In addition, we did not see the participants in a live interview, but again this has not been found to yield different patients characteristics when compared with studies on samples who have been assessed in-vivo [13]. The mean scores on the SAD measures at baseline were similar to previous studies [12], [13], [14], including an effectiveness study [16].

A second limitation concerns the control group and the use of a moderated online discussion forum. We did not check the activity in the discussion forum. It is known that far from all are active in such forums and that many may only watch and not post messages.

themselves [66]. While online discussion forums have been found to be beneficial in some studies [41], it is not well known if this is beneficial for persons with SAD even if no effects were found in a previous study [13]. We did not collect treatment credibility ratings and the control condition was most likely not perceived as an intervention for SAD.

A third limitation is that the knowledge test was developed and validated in association with this study and not independently. While the controlled design is a strength it is still possible that the knowledge test does not measure the most important aspects of SAD. In addition, the gains were modest in terms of raw score increases, but when we incorporated a rating of how certain the participant was about the response larger differences emerged. The clinical relevance of a modest gain in knowledge can be questioned even if certainty of knowledge may be more clinically relevant, However, certainty of knowledge without consideration of how correct that knowledge is would make no sense and hence we believe that the weighted scores are clinically relevant. We are also aware of the fact that the knowledge test had low internal consistency for the raw scores. This leads to lower statistical power and probably reflect the fact that the test was relatively easy and that certainty about the answers (with good reliability) was the factor we influenced by the treatment.

A fourth limitation concerns the therapists who were either categorized as experienced or inexperienced, which is a categorization that can be questioned. All therapists had basic training in CBT and the difference that emerged relating to the time taken to handle participants may represent a rapid learning curve. It is possible that relatively little experience is needed to learn how to guide clients in ICBT with much structure. In addition, while we had sufficient power to detect main effects of treatment the power to detect small effects of therapist experience was not sufficient. Moreover, our sample of persons with SAD and relatively high educational background may not be representative for patients seen in other settings for whom more experienced therapists may be needed to guide the programs.

### General Discussion

The mechanisms of change in ICBT for SAD are not well known, even if it is possible that cognitive aspects are involved as has been found in face-to-face CBT [67]. The ICBT tested in this trial includes both cognitive and behavioral components, but is based on a CBT model that underscores the importance of attention focused on the self, safety behaviors, and beliefs about social situations [68]. Mediators of change in ICBT for SAD have not been investigated and the specificity of the findings can be questioned as other treatments such as applied relaxation [12] and interpersonal psychotherapy [69] also lead to reduced symptoms even if they may be less effective than CBT.

The present study was the first to test if ICBT increased knowledge about SAD. This is an important topic as CBT incorporates psychoeducation with the aim to increase knowledge. However, correlations between knowledge gain and improvement on measures of SAD were small and it is likely that knowledge alone is not enough to reduce symptoms of SAD. Future research is needed to validate tests of knowledge regarding SAD and other conditions as it is unclear if lack of knowledge predisposes persons to develop and sustain SAD.

In spite of the limitation that the inexperienced therapists were trained psychologists, our study adds to the literature showing that guidance does not require much therapist experience [21], [35], [36]. In the previous studies only a few therapists have been involved. In contrast, the present study had several therapists being randomly assigned to guide the participants during treatment. More studies are needed to investigate the lower limits of competence in guided ICBT and also the conditions under which more expertise is called for.

### Conclusions

There are now several controlled trials showing that ICBT for SAD is effective. The present trial revealed that ICBT is better than only being offered to participate in an online discussion forum and that the effects are maintained one year later. Further, the study demonstrated that amount of previous therapist experience does not make a difference in terms of outcome but that experienced therapists may need less time to guide clients through the treatment. Finally, this is the first study to show that knowledge about social anxiety and its management actually increases with treatment.

## Supporting Information

Checklist S1 CONSORT Checklist
(DOC)

Protocol S1 Trial Protocol
(DOCX)
