# Supplementary material for: Therapist Experience and Knowledge Acquisition in Internet-Delivered CBT for Social Anxiety Disorder: A Randomized Controlled Trial
Source: PLoS One. 2012 May 23;7(5):e37411. doi: 10.1371/journal.pone.0037411 (PMC3359350; doi:10.1371/journal.pone.0037411)
Supplement: Protocol S1 — Trial Protocol (DOCX) [file pone.0037411.s002.docx]

**For professionals, the intended project / research plan (protocol) Part of ethics application translated from Swedish.**

Social phobia treatment via the Internet (SOFIE): Genetic variation and the effect of Internet delivered cognitive-behavioral therapy

**Background**

**Anxiety - a public health problem**

Anxiety can be described as an unpleasant feeling of fear, anxiety, tension or pressure. There is hardly anyone who has not experienced anxiety. Many people are troubled, however, of such reactions so often and so intensely that great suffering and limitations in life occurs, which is called anxiety disorders. In the U.S., population studies have shown that within one year 27 million people fulfil the criteria for at least one psychiatric disorder (1). Anxiety disorders are the most common psychiatric diagnosis in women and the second most common in men (2). In addition to suffering for individuals entails anxiety problems a considerable financial burden on society. The annual cost to society of these, such as in terms of sick leave and loss of earnings, is estimated at over 68 billion dollars in U.S. (1).

**Social phobia**

Epidemiological studies have shown that social phobia is the most common psychiatri syndrome in the Western world, with a lifetime prevalence of up to 10-15% (2-4). A person with social phobia is plagued by a fear of being audited or reviewed by others and ending up in the center of attention. Strong anxiety reactions can occur when the individual must perform something when other people, in particular strangers, watch, but also in normal social situations. Speaking before a group, attend meetings, go to a party or seek contact with other typical problem situations. Clinical studies have shown that persons with social phobia have a significantly impaired quality of life as well as problems with social relationships, partner relationships, well-being in the family, and functioning at work (5). Untreated social phobia often follows a chronic course (6) and often precedes other serious psychiatric disorders such as depression and alcohol problems. Social phobia is a widespread disease and also a gender specific problem as the symptoms are more common among women (4). Nausea is also common among the younger population, which may be related to the requirement to perform in social settings has increased in modern society (7). Social phobia has even been called a democracy problem because many victims do not dare to comment or participate in groups.

**Treatment of social phobia**

There are both psychological and pharmacological treatments that are proven effective in social phobia (8). Cognitive-behavioral therapy (CBT) is a structured psychological therapy that has been found to be effective in several controlled studies (8, 9). In CBT the therapist helps to change negative thoughts and beliefs that patients associate with different problem situations while the patient is exposed gradually to situations that arouse anxiety. We have in the past five years developed an Internet-delivered CBT program that has been tested in randomized trials (10-12). These studies show improvements that are fully comparable with those seen in regular face-to-face CBT (9). In the Internet- delivered CBT program the patient works with a self-help manual (13) made available through the Internet, while e-mail correspondence is weekly with a therapist (in a secure system). Patients also have access to an online discussion forum and the program can also be combined with exposure exercises carried out during the group meetings (10). In addition to studies on social phobia (the so-called SOFIE projects), the research also showed similar positive treatment outcomes of Internet-based treatments of panic disorder, tinnitus, chronic pain, depression, bulimia / binge eating disorder, and compulsive gambling.

**Genetic markers and treatment**

A heritability rate of over 30% has been reported in twin studies of social phobia (14). This suggests that genes have an important role in the onset of problems, even though we have no detailed knowledge of how genes, or gene interaction with brain function and environment, contribute to social anxiety. Serotonin transporter gene is a potentially important gene for social phobia. The anxiolytic effects of selective serotonin reuptake inhibitors (SSRIs) appear to be mediated by an inhibition of the serotonin transporter, ie. the protein that återtransporterar serotonin into the presynaptic neuron (15). Transportation of serotonin is largely influenced by genetic factors and there are related differences in the resumption of short and long alleles in the serotonin transporter gene promotor region. People who are homozygous for the long allele (l / l) has an increased reuptake of serotonin compared to those who are carriers of one or two short (s / l or s / s) alleles (16). Several studies have shown that the short allele is associated with higher general anxiety and neural activity in the amygdala (17), ie. a brain region that is strongly associated with emotional reactions such as fear and anxiety (18,19).

We have previously reported that people with social phobia who carry the short allele have a more symptoms of social anxiety, and an increased reactivity in the right amygdala during social anxiety provocation (20). A recent treatment study showed that variation in the serotonin transporter gene was important for how well patients with social phobia responded to treatment with serotonergic drugs (21). We also have new data suggesting that tryptofanhydroxylas-2 (TPH2) gene, coding for serotonin synthesis in the brain, is related to the placebo response in social phobia (22), as well as the amygdala activity (22.23). The TPH2 polymorphism we studied is called the G-703T where the T allele (risk allele) compared to G allele (22.23)

**Purpose**

Currently, it is not known if genetic variation is important in psychotherapeutic treatments such as CBT. This project aims to, in addition to a controlled evaluation of Internet-based CBT, identify if particular serotonin-related genes affect how people with social phobia respond to Internet-based CBT.Since there are few studies that have examined what predicts treatment outcome in social anxiety disorder, it is theoretically and clinically important to study this as well. In the trial we will also develop and include a test of knowledge about social phobia and test the role of therapist experience. This is not described further here as it is not a main ethical consideration.

**Issues**

The project's primary issues are:

1. Is the effect of Internet-based CBT influenced by variation in the serotonin transporter and the TPH2 genes? More specifically,

a) How is the short and long alleles in the promoter region of the serotonin transporter gene related to CBTresponse?

b) How is this related the T and G alleles of the G-703T polymorphism related to CBT response?

2. How the above mentioned genetic variations related to symptom profile at social phobia (before treatment), More specifically,

a) the subset of social phobia (generalized or non-generalized social phobia)

b) severity as measured by social phobia scales

c) the degree of general anxiety as measured by questionnaire

d) the degree of depression measured by questionnaire

e) influence quality of life as measured by questionnaire

Gene analyzes focusing initially on the variation in the serotonin transporter-and TPH2 genes, but also other genes will be evaluated in the exploratory analysis. These will be limited to genes that directly or indirectly affect neurons with the importance of cognitive or emotional reactions (eg those that use dopamine, glutamate, GABA, vasopressin, acetylcholine or norepinephrine as a neurotransmitter).

The project will also to evaluate whether the Internet-based CBT program is effective when compared against an active control group (randomized). In addition to the project study additional predictors of treatment outcome. Previous research has, among other things identified the severity of social anxiety before treatment influenced the outcome. The project will also evaluate the two hitherto unstudied factors that may influence treatment outcome, namely the acquired knowledge (psychoeducation) and therapeutic alliance. The latter predictors will be measured by questionnaire.

**Design**

Two hundred and fifty people diagnosed with social phobia in an experimental study be randomized into two treatment groups: 1) Internet-based CBT (n = 125) or 2) Control group who participate in a moderated discussion forum on the web (n = 125). The control group will be treated with Internet-based CBT after the initial group has completed their treatment, ie. after nine weeks. Genotyping based on saliva samples is done before starting treatment for those who leave the voluntary sample.

**Procedure**

This study will be conducted at Uppsala University (Tomas Furmark, PhD., Project Manager, Mats Fredrikson professor, Lisa Ekselius professor, consultant) in collaboration with Professor Gerhard Andersson and licensed psychologist / PhD Per Carlbring at Linköping University, and Professor Elias Eriksson University of Gothenburg.

**Subjects**

The number of volunteers is estimated at 250 people, 125 in each group. The choice of the number of people based on 125 people in each group is assumed, based on power calculation, be sufficient for this to be a reasonable chance to detect clinically relevant effects if any. The research group's previous genetic analysis of social anxiety disorder have found clinically significant effects detected in even smaller samples (20,22,23), but with 125 people in each group we will have a fair chance to detect effects of gene variants (and gene-gene interactions) on treatment response and symptom profile. The subjects will be recruited through the project's website ([www.studie.nu](https://mail.liu.se/owa/redir.aspx?C=c770dbacb4d543a68289873d76df6c57&URL=http%3a%2f%2fwww.studie.nu)), and via a link on the Anxiety Disorder Society's website. The potential subject will be able to register interest on the study website after taking part of the information (informed consent). It informs also the subject of ethical research principles including that participation is voluntary and that at any time can stop the current treatment. Only persons aged 18 and above will be included.

**Screening, pretreatment assessment and randomization**

The screening process will follow the procedures from our recent study in the SOFIE project (No. 2005:187). When the subject has expressed an interest, a code is sent out which gives access to the portion of the site on which the screening instruments available. The participant answers the first forms: Social Phobia Screening Questionnaire-SPSQ (3) and Montgomery Asberg Depression Rating Scale-MADRS (24). Participants must meet the criteria for social phobia using the international classification system Diagnostic and Statistical Manual of Mental Disorders, 4th edition (DSM-IV) (25). This is mainly determined by SPSQ form that has been shown to have good sensitivity and specificity in comparison with the structured diagnostic interviews. In addition, we include a telephone screening, which follows a structured clinical interview, Structural Clinical Interview for DSM-IV - Axis I Disorders: SCID I (26). Individuals with current major depression and / or suicide-prone persons will be excluded from the study. Furthermore, the subject will be asked to submit a signed consent form to the research group. The constent form involves consent to do genetic analyzes and stores saliva samples in a biobank, the processing of personal data concerning him / her in accordance with the Data Protection Act and that he / she hs received information on the website about how the study.

The subjects meeting the inclusion criteria will then be randomized into two groups so that half may begin directly with the Internet-based self-help treatment program (treatment cycle 1) while the other half gets allocated to the control group which involves participation in a moderated discussion forum on the Internet for nine weeks after which they are treated as usual with the Internet-based program (treatment session 2). The latter group controls in this manner for the time and measurement.

Before the treatment groups may have access to self-help program will fill the subjects in the pretreatment measures consisting of: Social Interaction Anxiety Scale (27), Social Phobia Scale (27), Liebowitz Social Anxiety Scale-LSAS (28), Beck Anxiety Inventory (29), and Quality of Life Inventory (30). All these forms are internationally established and have been shown to have good psychometric properties. Two handwritten forms, which measures the degree of alliance respectively. knowledge acquisition will also be designed and administered during the study period.

**Treatment**

Treatment will be through the Internet in two rounds, and will take advantage of our previously developed structured self-help program that lasts for a total of nine active treatment weeks. The guided self-help program is based on an updated version of the previously used in our approved studies (Ref: 02-555, 03-636, 2004: m-163, 2005:187) ie. on well-proven principles from cognitive behavioral therapy. Following each treatment week participants will be asked a number of questions and send answers via the website to show that they have assimilated the module content. In connection with this they will obtain the password to the next module. Each week, participants also fill in LSAS form and question 9 from MADRS dealing with the zest for life and possible suicidal thoughts. This weekly measurement helps to monitor participants' current state and intervene if needed. All participants will be participate in Internet discussion group where they can speak and send messages to each other. The different treatment groups will have separate discussion groups, which are monitored daily. The degree of anonymity determines the participants themselves, and they may for example choose the username of your choice. The study coordinators will be available to answer questions.Psychology Candidates during the final phase of their education will, according to a well-established model, participate in the project in connection with their MSc theses. As in our previous treatment studies (10-12) they will serve as e-mail therapists under supervision. We have previously evaluated the efficacy of CBT program administered in this way in five randomized controlled trials, of which three have so far been published (10-12) along with an open study (31).

**Genetic analyzes**

Each participant will be asked to provide a voluntary saliva sample which is then analyzed with respect to the previously mentioned polymorphisms. Participants will be sent a coded test tube which is adapted for mail handling (Oragene, see [http://www.dnagenotek.com](https://mail.liu.se/owa/redir.aspx?C=c770dbacb4d543a68289873d76df6c57&URL=http%3a%2f%2fwww.dnagenotek.com)) subsequently videaresänds to Professor Elias Eriksson of Gothenburg University for analysis. Information about individuals' identities will not be enclosed these samples. Saliva samples will be stored in a biobank in the pharmacological department, the Department of Neuroscience and Physiology, Sahlgrenska Academy, Göteborg University, which also analyzes will be carried out. The management of the genetic material will follow our previous genetic studies of social phobia which has been approved by the Ethics Committee (see, eg, Ref 2004: M-416 and 421-2007-2223, the latter from the ethical review board in Stockholm).

**Evaluation**

Posttreatment measurements are made in connection with the final module completed. Follow up with a short phone interview and form occurs after 12 and 24 months. The latter is done to evaluate the longer-term treatment effects and to compare these with the short-term effects. Pre-, post-and follow-up measurements will be made with established international form (see above). An attempt will be made to try to identify predictors of treatment success and factors that may predict which individuals this type of treatment is best suited for.

**Schedule**

A total period of 6 months (August 2008 - January 2009) will be required for the selection, diagnosis, management of forms and saliva samples, treatment and initial analysis. The project will provide large amounts of data. In-depth analysis, as well as the compilation and writing of scientific reports, and will therefore last for a long time.

**Security**

The participant is recommended to get a free e-mail address with "Hushmail" that offers encryption (2048 bit) of the email. The service is operated by the company Hush Communications. All e-mails sent from Hushmail is kryperad and can not be traced. The encrypted e-mail correspondence will be via password-protected computers with topical definitions of antivirus software. The correspondence with clients will be printed and stored in purpose-designed covers. These, and other materials, will be tagged with an ID number and stored in a locked filing cabinet. When the retention period, according to the adopted standard, expired material will be destroyed. Data from the questionnaires will be recorded in anonymous form. This means that participants in the trials can not be identified afterwards. The results will also be analyzed at the group level.

**Privacy and genetic analyzes**

To protect participants' identity will be samples and data to be encoded. All information will be kept strictly confidential and in accordance with the Data Protection Act. Code list, through which tube number will be linked to individual, will be kept locked up in the project at Uppsala University. The results of the analysis disclosed not to any other party, unless required by operation of law. Although the participant's family, doctors, insurers and employers included in the concept of "another party". The genetic results obtained are thus only for research. Participants may request a copy of analysreslutaten, as well as correction or that the sample be destroyed.

No individual data is traceable in the scientific reports presented from this study and the results will be reported as averages. The participant's identity can not be tracked by the results that are stored but this can only be identified with a special code. The samples will be stored for up to 15 years and then destroyed. Samples will not be used for any purpose other than that described here

**Ethical considerations**

The subjects participating voluntarily in the study. The study assumes that participants are informed that in the case of self-help any improvement requires active engagement.

The following ethical risks have been identified: the first is that participants in need of specialized medical investigations may be included. At the slightest suspicion of unclear etiology, participants will be asked to seek medical advice in the community. We will also provide information about self-help program is not suitable if you suffer from severe depression or suicidal thoughts.

The people who are excluded from the study may perceive this negatively. All individuals who are excluded will therefore have a recommendation on how and where to seek alternative treatment in the community, and in cases it deems appropriate, make recommendations on appropriate self-help literature. Participants will be randomly allocated to one of the two groups (treatment cycle 1 and 2). To provide the best scientific evidence is not allowed participants to choose a group and those who do not agree to this will therefore not be included in the study.

Participants in the control group had to wait for their treatment in nine weeks until the first group therapy is completed, which may be perceived as negative. While these individuals will be treated with Internet-based CBT and during the waiting time access to a moderated discussion forum.

To submit a saliva sample for genetic analysis can be seen as a possible privacy violation. They participate voluntarily and even participants who do not want to leave the test will be able to participate in treatment. Participants advised that anyone who works with the samples have to keep research data and results secret. Participants are informed of the results of the assays are not disclosed to any other party, including family, doctors, insurers or employers, unless required by operation of law. Information is also given that the genetic results are only for research and not used for any other purpose. As noted, all information will be kept absolutely confidential and the participant is assigned a personal code. Survey participants are offered ongoing opportunity to get answers to questions by telephone and encrypted e-mail during the study.

**Previous experience**

Extensive experience in similar studies in the project for both Internet-based therapy (10-13, 31) that genetic analyzes (20,22,23) of people suffering from social phobia.

**Significance**

The project can make important contributions to our understanding of how psychological treatment for SAD works. A particularly important question concerns the possibility of predicting treatment response based on genetic variation, which can have significant treatment implications. The results of the project will be significant for the understanding of social phobia, but also for other psychiatric disorder and mood disorders like depression where anxiety is an essential part of the symptomatology.

**References**

1. Greenberg PE, Sisitsky T, Kessler RC *et al.* The economic burden of anxiety disorders in the 1990s. *J Clin Psychiatry* 1999;60:427-435.
2. Kessler RC, McGonagle KA, Zhao S *et al.* Lifetime and 12-month prevalence of DSM-III-R psychiatric disorders in the United States. *Arch Gen Psychiatry* 1994;51:8-19.
3. Furmark T, Tillfors M, Everz P.O. et al. Social phobia in the general population: Prevalence and sociodemographic profile. *Soc Psychiatry Psychiatric Epidemiol* 1999;34:416-424.
4. Furmark T. Social phobia: Overview of community surveys. *Acta Psychiatr Scand.* 2002;105:84-93.
5. Wittchen HU, Fuetsch M, Sonntag H *et al.* Disability and quality of life in pure and comorbid social phobia. Findings from a controlled study. *Eur Psychiatry* 2000;15:46-58.
6. Yonkers KA, Dyck IR, Keller MB. An eight-year longitudinal comparison of clinical course and characteristics of social phobia among men and women. *Psychiatr Serv.* 2001;52:637-643.
7. Heimberg RG, Stein, MB, Hiripi E, Kessler RC. Trends in the prevalence of social phobia in the United States: a synthetic cohort analysis of changes over four decades. *Eur Psychiatry* 2000;15:29-37.
8. SBU - Statens beredning för medicinsk utvärdering. *Behandling av ångestsyndrom, vol 1 & 2.* Mönlycke: Elanders Infologics Väst AB, 2005.
9. Fedoroff IC, Taylor S. Psychological and pharmacological treatments of social phobia: a meta-analysis. *J Clin Psychopharmacol*. 2001;21:311-324.
10. Andersson G, Carlbring P, Holmström A, *et al.* Internet-based self-help with therapist feedback and in-vivo group exposure for social phobia: a randomized controlled trial. *J Consult Clin Psychol.* 2006;74: 677-686.
11. Carlbring P, Gunnarsdóttir M, Hedensjö L *et al.* Treatment of social phobia from a distance: A randomized trial of internet delivered cognitive behavior therapy (CBT) and telephone support. *Br J Psychiatry.* 2007;190:123-128.
12. Tillfors M, Carlbring P, Furmark T, *et al.* Treating university students with social phobia and public speaking fears: Internet delivered self-help with or without live group exposure sessions. *Depr & Anxiety,* in press.
13. Furmark T., Holmström A, Sparthan E. *et al.* *Social fobi. Effektiv hjälp med kognitiv beteendeterapi.* Stockholm: Liber, 2006
14. Kendler KS, Neale MC, Kessler RC, *et al*. The genetic epidemiology of phobias in women. *Arch Gen Psychiatry* 1992;49:273-281.
15. Meyer JH, Wilson AA, Ginovart N, *et al.* Occupancy of serotonin transporters by paroxetine and citalopram during treatment of depression: A [11C]DASB PET imaging study. *Am J Psychiatry* 2001;158:1843-1849.
16. Lesch KP, Bengel D, Heils A, *et al.* Association of anxiety-related traits with a polymorphism in the serotonin transporter gene regulatory region. *Science* 1996;274:1527-1531.
17. Hariri AR, Mattay VS, Tessitore A, *et al.* Serotonin transporter genetic variation and the response of the human amygdala. *Science* 2002;297:400-403.
18. LeDoux JE. Emotion circuits in the brain. *Annu Rev Neurosci* 2000;23:155-184.
19. Whalen PJ. Fear, vigilance and ambiguity: Initial neuroimaging studies of the human amygdala. *Current Dir Psychol Sci.* 1998;7:177-188.
20. Furmark T, Tillfors M, Garpenstrand H, *et al.* Serotonin transporter polymorphism related to amygdala excitability and symptom severity in patients with social phobia. *Neurosci Lett*. 2004;362:189-192.
21. [Stein MB](http://www.ncbi.nlm.nih.gov/entrez/query.fcgi?db=pubmed&cmd=Search&itool=pubmed_Abstract&term=%22Stein+MB%22%5BAuthor%5D), [Seedat S](http://www.ncbi.nlm.nih.gov/entrez/query.fcgi?db=pubmed&cmd=Search&itool=pubmed_Abstract&term=%22Seedat+S%22%5BAuthor%5D), [Gelernter J](http://www.ncbi.nlm.nih.gov/entrez/query.fcgi?db=pubmed&cmd=Search&itool=pubmed_Abstract&term=%22Gelernter+J%22%5BAuthor%5D). Serotonin transporter gene promoter polymorphism predicts SSRI response in generalized social anxiety disorder. [*Psychopharmacology*](javascript:AL_get(this,%20'jour',%20'Psychopharmacology%20(Berl).');)  2006;187:68-72.
22. Furmark T, Appel L, Henningsson S, *et al*. A link between serotonin-related gene polymorphisms, amygdala activity and placebo-induced anxiety relief. *Submitted*.
23. Furmark T, Henningsson S, Appel L, *et al*. Genotype over diagnosis in amygdala responsiveness: Affective processing in social anxiety disorder. *J Psychiatry Neurosci.* in press.
24. Svanborg P, Åsberg M. A new self-rating scale for depression and anxiety states based on the comprehensive psychopathological rating scale. *Acta Psychiatrica Scand.* 1994; 89: 21-2
25. American Psychiatric Association. *Diagnostic and statistical manual of mental disorders - Text Revision* (4 ed.). Washington, DC, USA: American Psychiatric Press, 2000.
26. First MB, Gibbon M, Spitzer RL, Williams JBW. *Structured clinical interview for DSM-IV Axis I Disorders (SCID-I).* Washington, D.C.: American Psychiatric Press, 1997.
27. Mattick RP, Clarke JC. Development and validation of measures of social phobia scrutiny fear and social interaction anxiety. *Behav Res Ther.* 1998; 36: 455-470.
28. Liebowitz MR.. Social phobia. *Mod Prob Pharmacopsychiatry,* 1987;22:141-173.
29. Beck, AT, Epstein N, Brown G, Steer RA. An inventory for measuring clinical anxiety: Psychometric properties. *J Consult Clin Psychol.* 1988;56: 893-897.
30. Frisch MB, Cornell J, Villanueva M, Retzlaff PJ. Clinical validation of the Quality of Life Inventory. A measure of life satisfaction for use in treatment planning and outcome assessment. *Psychol Assessment* 1992; 4: 92-101.
31. Carlbring P, Furmark T, Steczkó J, *et al*. An open study of Internet-based bibliotherapy with minimal therapist contact via e-mail for social phobia. *Clin Psychologist* 2006;10:30-38.
